# 

**Published:** 1891-12

**Authors:** 


					﻿io cts. a copy.
lj ll ll ivid c.n, io »i.
jji.uo uei ky ecu.
WALLS*
,|'AI(\\|-
WEAITW
ESTABLISHED 18B4
U°±-38.
c/H- 12.
UPTOWN OFFICE. 840 WEST 59th STREET.
Entered as Second-Class Matter at the Post-Office, New York.
				

